# Preliminary real‐world trends in outpatient revenue following implementation of disease‐modifying therapies in general hospital psychiatry in Japan

**DOI:** 10.1002/pcn5.70328

**Published:** 2026-04-13

**Authors:** Kyohei Otani, Hiroyuki Ishihara, Tetsu Nakamura, Kana Kawabe

**Affiliations:** ^1^ Department of Psychiatry Kakogawa Central City Hospital Hyogo Japan; ^2^ Department of Neurology Kakogawa Central City Hospital Hyogo Japan; ^3^ Department of Diagnostic Radiology and Interventional Radiology Kakogawa Central City Hospital Hyogo Japan; ^4^ Administration Department Kakogawa Central City Hospital Hyogo Japan

## Abstract

Implementation of anti‐amyloid disease‐modifying therapy (DMT) within a psychiatry‐led dementia care program enabled structural transformation of service delivery in a general hospital setting. A multidisciplinary model integrating psychiatry, neurology, and radiology facilitated coordinated care and safe treatment implementation. This approach was associated with increased outpatient revenue per patient without a corresponding increase in workforce burden, suggesting that high‐value neuropsychiatric treatments can shift psychiatry from a cost center to a revenue‐generating service.

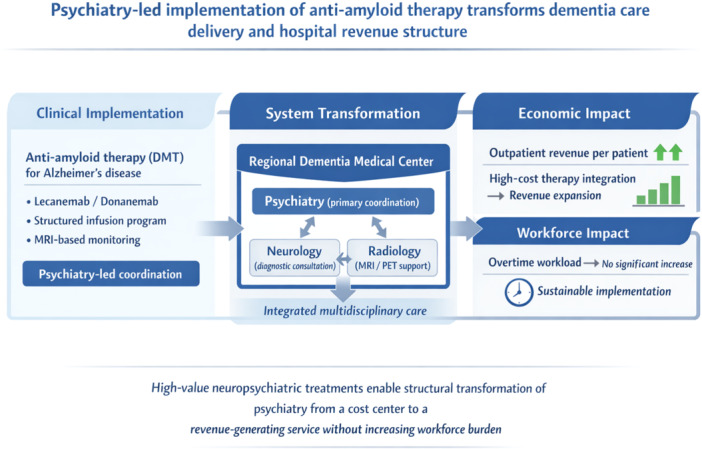

Disease‐modifying therapies (DMTs) for Alzheimer's disease, including anti‐amyloid monoclonal antibodies, have recently entered routine clinical practice in Japan.^1^, ^2^ Unlike many Western healthcare systems in which dementia care is primarily managed by neurology departments, psychiatrists in Japanese general hospitals frequently play a central role in dementia diagnosis and longitudinal treatment.^3^ However, real‐world evidence describing how implementation of DMTs affects outpatient revenue structures in general hospital psychiatry remains limited. Although psychiatric departments involved in DMT implementation may conduct more dementia differential diagnoses,^4^ the economic implications of this expanded clinical role remain poorly understood amid growing concerns regarding financial sustainability in Japanese psychiatry^5^, ^6^, ^7^, ^8^ and broader challenges in mental health service reimbursement.

We conducted a retrospective descriptive analysis of aggregated administrative outpatient revenue data from the Department of Psychiatry at Kakogawa Central City Hospital (Table 1). Data were extracted from the hospital's administrative dashboard for fiscal years (FY) 2022–2025 (April 2022 to October 2025). Revenue per visit was calculated separately for initial visits, follow‐up visits, and total outpatient activity.

**Table 1:** Annual outpatient revenue per visit.

Fiscal year	Initial visit	Follow‐up visit	Total per visit
FY2022	16,768 (US$117)	5148 (US$36)	5560 (US$39)
FY2023	16,351 (US$114)	6013 (US$42)	6429 (US$45)
FY2024	19,207 (US$134)	7948 (US$55)	8448 (US$59)
FY2025^a^	21,949 (US$153)	13,240 (US$92)	13,401 (US$93)

*Note*: Exchange rate: 1 USD = 143.5 JPY (approximate average for the study period). Study setting: Kakogawa Central City Hospital, a 600‐bed public general hospital with 34 clinical departments providing secondary emergency care in Hyogo Prefecture, operating a Regional Dementia Medical Center under psychiatric direction. Disease‐modifying therapy (DMT) administration coordinated primarily by psychiatry in collaboration with neurology and radiology departments. The Department of Neurology provides consultation for neurological assessment, differential diagnosis of cognitive impairment, and evaluation of neurological symptoms during treatment monitoring.

Abbreviation: FY, fiscal years.

^a^ FY2025 includes April–October 2025 (partial year), preceding November 2025 drug pricing revision.

During the study period, lecanemab was introduced in late FY2023, with systematic scaling during FY2024–2025. Other high‐value neuropsychiatric treatments—including calcitonin gene‐related peptide‐targeted migraine therapy^9^ and long‐acting injectable antipsychotics^10^—were also implemented. The present analysis does not attempt to attribute revenue changes to any single therapeutic intervention. No additional full‐time psychiatrists or nurses were added during the observation period.

Total outpatient revenue per visit increased 141% from FY2022 to FY2025 (Table 1). Year‐over‐year growth accelerated progressively (15.6%, 31.4%, and 58.6%). Initial visit revenue increased modestly (JPY 16,768 to JPY 21,949; +31%), whereas follow‐up visit revenue increased substantially (JPY 5148 to JPY 13,240; +157%), suggesting that longitudinal treatment monitoring contributed more to revenue growth than diagnostic evaluation alone.

Annual outpatient visit volume remained relatively stable (FY2022: 11,203; FY2023: 10,100; and FY2024: 10,223), indicating that increased revenue per visit—rather than increased patient numbers—primarily drove growth. The modest decline in FY2023 likely reflects a hospital‐wide reduction in part‐time physician staffing during this period.

Several limitations warrant consideration. First, this single‐center descriptive analysis from a public general hospital operating a Regional Dementia Medical Center may not generalize to smaller facilities, rural settings, or institutions without specialized neuroimaging capabilities. Second, while revenue expansion temporally coincided with DMT implementation, other concurrent structural and reimbursement changes may also have contributed and cannot be excluded. Third, although gross revenue increased substantially, this metric alone does not reflect medication acquisition costs, imaging‐related expenditures, or personnel burden. Moreover, subsequent revenue structures may differ following the November 2025 drug pricing revisions. Mean monthly overtime hours among psychiatrists remained stable across the observation period (FY2023: 14.4 h; FY2024: 15.7 h; and FY2025: 11.3 h), as did overtime among outpatient nursing staff (FY2023: 5.9 h; FY2024: 4.6 h; and FY2025: 5.3 h), suggesting that revenue growth was not associated with a substantial increase in recorded overtime hours. However, detailed cost accounting and treatment‐specific margin analysis were beyond the scope of this preliminary report.

These findings suggest that, within the Japanese healthcare context—where psychiatrists commonly provide comprehensive dementia care through Regional Dementia Medical Centers—the implementation of DMTs may meaningfully alter outpatient revenue structures without expansion of staffing or patient volume. Nevertheless, whether such revenue transformation translates into sustainable net profitability remains uncertain.

Future research incorporating treatment‐specific revenue attribution, detailed cost analysis, and patient‐level data will be essential to determine the long‐term economic sustainability of DMT implementation in general hospital psychiatry.

## AUTHOR CONTRIBUTIONS

K.O. conceived the study, analyzed the data, and drafted the manuscript. H.I. contributed to clinical data interpretation regarding dementia and reviewed the manuscript. T.N. provided imaging‐related insights and reviewed the manuscript. K.K. provided administrative revenue data and reviewed the manuscript. All authors approved the final version.

## CONFLICT OF INTEREST STATEMENT

K.O. has received speaker honoraria from Eisai, Eli Lilly, Daiichi Sankyo, and Otsuka Pharmaceutical within the past 12 months. Others declare no conflicts of interest.

## ETHICS APPROVAL STATEMENT

According to the policy of the Institutional Review Board of Kakogawa Central City Hospital, ethical review was not required for this retrospective analysis of de‐identified, anonymized administrative data without direct patient contact.

## PATIENT CONSENT STATEMENT

Not applicable. This study utilized anonymized, non‐identifiable administrative data without individual patient information.

## CLINICAL TRIAL REGISTRATION

Not applicable. This was a retrospective observational study using administrative data and was not registered as a clinical trial.

## Data Availability

Aggregated administrative data are available from the corresponding author upon reasonable request.
